# An Acellular Platform to Drive Urinary Bladder Tissue Regeneration

**DOI:** 10.1002/adtp.202400158

**Published:** 2024-12-20

**Authors:** Mitali Kini, Matthew I. Bury, Arun K. Sharma

**Affiliations:** Department of Urology, Feinberg School of Medicine, Northwestern University, Chicago, IL 60611, USA; Division of Pediatric Urology, Department of Surgery, Ann & Robert H. Lurie Children’s Hospital of Chicago, Chicago, IL 60611, USA; Center for Regenerative Nanomedicine, Northwestern University, Chicago, IL 60611, USA; Department of Urology, Feinberg School of Medicine, Northwestern University, Chicago, IL 60611, USA; Division of Pediatric Urology, Department of Surgery, Ann & Robert H. Lurie Children’s Hospital of Chicago, Chicago, IL 60611, USA; Center for Regenerative Nanomedicine, Northwestern University, Chicago, IL 60611, USA; Department of Biomedical Engineering, McCormick School of Engineering, Northwestern University, Evanston, IL 60208, USA

**Keywords:** cell-free therapy, tissue regeneration, urinary diversion, wound healing

## Abstract

Impaired bladder compliance secondary to congenital or acquired bladder dysfunction can lead to irreversible kidney damage. This is managed with surgical augmentation utilizing intestinal tissue, which can cause stone formation, infections, and malignant transformation. Co-seeded bone marrow mesenchymal stem cell (MSC)/CD34+ hematopoietic stem cell (HSPC) scaffolds (PRS) have been successful in regenerating bladder tissue. However, the acquisition of viable cells is challenging in the clinical setting. Here, the regenerative capacity of human MSC/CD34+ co-cultured total condition media (TCM) is compared to media alone in immune-competent rats augmented with PRS following partial cystectomy. Augmented bladders are instilled with media (control, *n* = 4) or TCM (*n* = 5) twice a week for 4 weeks. Regenerated tissue is analyzed for smooth muscle, urothelium, vascular, and peripheral nerve regrowth. Urodynamic (UDS) measures are performed pre- and 4 weeks post-augmentation. The results demonstrate that TCM-instilled grafts have greater muscle content, larger average urothelial widths, higher percent vascularization, and more robust neural infiltration post-augmentation. UDS demonstrates greater percent bladder recovery in the TCM group, indicating functional improvement in bladder storage capacity. This study is the first to propose the use of cell-free TCM as an alternative to traditional cell-seeded scaffolds to promote bladder tissue regeneration.

## 1. Introduction

Urinary bladder dysfunction can arise due to congenital or acquired disease or trauma in both adult and pediatric patients. Neurogenic etiologies include spina bifida or spinal cord injury, which leads to dysregulated detrusor muscle contraction and subsequent bladder decompensation. This may also occur in patients with long-standing bladder outlet obstruction, radiation cystitis following bladder cancer treatment, and military personnel subjected to battlefield injuries of the pelvis. While conservative medical management is possible in some patients with dysfunctional bladders, many patients progress to end-stage disease in which the bladder is unable to adequately expand during filling, and unable to contract sufficiently to empty. This poor compliance can lead to permanent upper urinary tract deterioration.^[1]^ Surgical augmentation enterocystoplasty has traditionally been employed as a last-resort treatment in these patients. This procedure involves the excision of a segment of small bowel, which is then used to augment the bladder thereby increasing capacity and compliance in order to re-establish low storage pressures.^[2,3]^ In instances in which an end-stage bladder cannot be salvaged due to poor tissue quality, or patients are unable to manage bladder maintenance with intermittent catheterization and irrigation, incontinent urinary diversion in the form of an ileal conduit may be offered following cystectomy.^[2,4]^ However, the use of intestinal tissue in both procedures can lead to several complications including metabolic derangements, infections, stone formation, and risk of malignant transformation. Patients are often required to catheterize several times a day to avoid excessive mucus production and urinary tract infections, which can lead to a diminished quality of life. In the pediatric population, regular urethral catheterization and bladder irrigation can be a challenge due to patient intolerance. This can lead to mucus buildup, infectious stone formation, risk of urosepsis, and a potential need for additional surgical interventions throughout these patients’ lifetimes.^[5]^

The field of regenerative engineering has aimed to create functional bladder substitutes ex vivo in order to reduce the morbidity associated with the use of bowel in bladder augmentation surgery. In recent years, ongoing strategies to reconstruct bladder tissue have used pliable scaffolds in combination with well-characterized stem cell populations. Work from our group has characterized elastic POC [poly(1,8 octamethylene citrate)] scaffolds (also known as the CystoSTEM platform) co-seeded with human spina bifida derived bone marrow (BM) mesenchymal stem cells (MSCs) and donor matched CD34+ hematopoietic stem/progenitor cells (HSPCs), which have been shown to act synergistically in a nude rat bladder augmentation model.^[6]^ Autologous MSCs and CD34+ HSPCs have previously been shown to have favorable proregenerative properties and in former animal studies, dual-seeded scaffolds demonstrated improved efficacy in bladder tissue regeneration compared to scaffolds seeded with single cell populations (MSC or CD34+ HSPCs alone). Results from these rodent models have paved the way for studies comparing cell-seeded poly(1,8-octamethylene-citrate-co-octanol) scaffolds (PRS) to autologous ileum in a baboon bladder augmentation model.^[7,8]^ This scaffold construct not only possesses bladder-like mechanical properties, but is biodegradable and highly tunable, with enhanced elasticity confirmed by tensile stress/strain testing and improved suturability compared to the prior POC scaffold design. Data from both the rodent and baboon models using PRS with cellular seeding have confirmed robust regeneration of endogenous peripheral nerves, smooth muscle, and urothelial tissue and evidence of increased blood vessel density throughout the entirety of the augmented PRS grafts, as well as improvements in urodynamic parameters, including decreased storage pressures and increased bladder compliance.^[7,8]^ However, the acquisition of these bone marrow-derived stem cell populations via bone marrow aspiration poses significant costs and morbidity for patients. Additionally, the downstream propagation of these cells while maintaining cell viability is challenging and resource-intensive. In this study, we demonstrate that bladder instillation of total condition media (TCM) obtained from the co-culture of MSCs and CD34+ HSPCs can promote tri-layer tissue regeneration in the absence of cell seeding. This approach offers a completely cell-free alternative to promoting bladder regeneration in patients augmented with an elastic synthetic scaffold (PRS) thereby obviating the need for cell acquisition or long-term cell culture. Here, we compare the bladder tissue regenerative capacity of human MSC/CD34+ HSPC co-cultured TCM to media alone in an immune-competent rat bladder augmentation model in which rats underwent partial cystectomy and were augmented with an unseeded PRS graft. This study is the first to demonstrate robust bladder tissue regeneration following scaffold augmenttation using an entirely cell-free intravesical instillation derived from MSCs and CD34+ HSPCs.

## 2. Results

### 2.1. Blood Vessel Quantification and Microvasculature Staining

**Figure 1.**
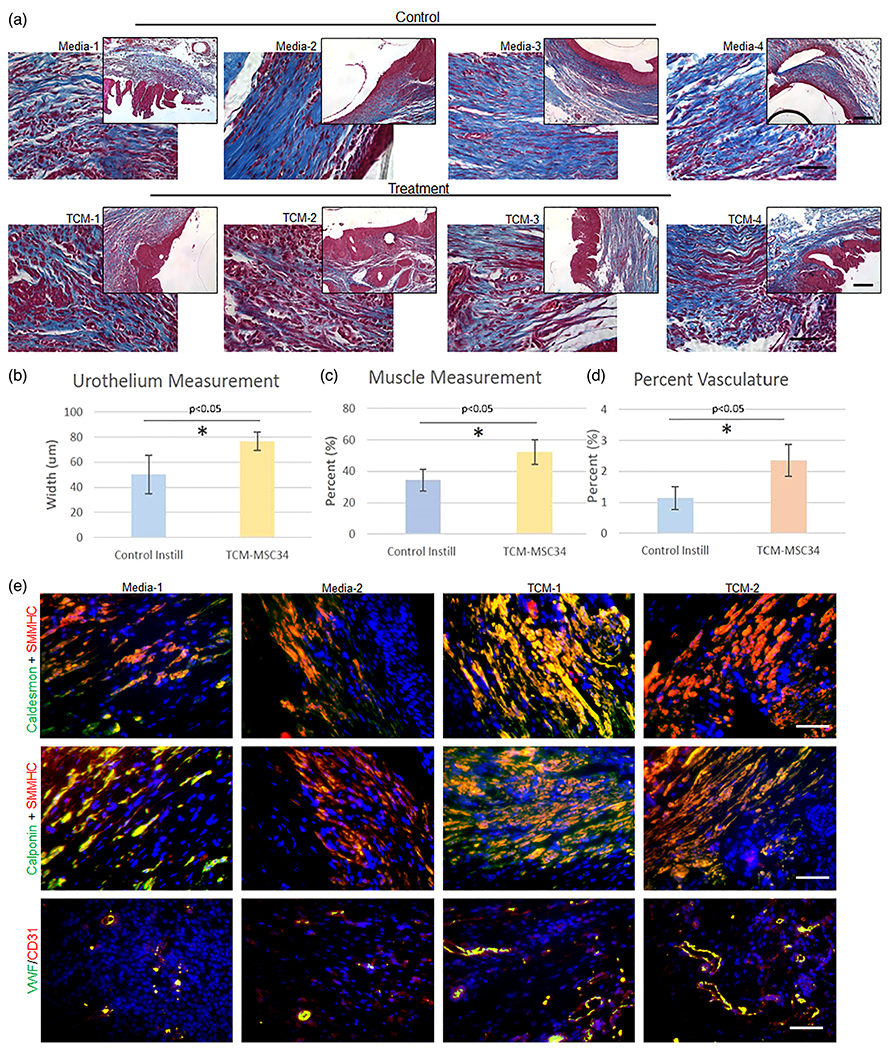
Bladder smooth muscle regeneration, urothelium, and vasculature asment. A) Trichrome staining of bladder tissue in control and Total condition media (TCM) animals. B) Urothelium width μm) was manually measured and quantified. Data demonstrate TCM group had significantly greater average urothelial width than control group. C) Muscle:collagen content was manually quantified. TCM group had significantly greater percent muscle content than control group. D) Number and size of blood vessels in each image were manually measured and quantified. TCM group demonstrated significantly greater percent vasculature compared with control group. E) Immunofluorescence co-staining of Caldesmon (green) + Smooth muscle myosin heavy chain (SMMHC) (red) and Calponin (green)+ SMMHC demonstrate greater overall protein expression in TCM regenerated tissue when compared to the control group. Immunofluorescence co-staining of von Willebrand factor (vWF) (green) and CD31 (red) in the third row demonstrates greater expression in the TCM tissue compared to control group. *p* < 0.05 was considered statistically significant. Data represents means ± SE. Inset images 10X scale bar set to 500 μm, 40X Scale bars represent 50μm.

Blood vessel formation is a crucial component of bladder tissue regeneration, as this ensures adequate oxygenation and nutrient delivery to the graft. We quantified blood vessel formation in all experimental and control groups following tissue processing (Figure 1A). Mean vessel number in regenerated tissue was similar between the TCM-instilled and control animal groups (82.5 ±7.6 vessels mm^−2^ versus 75.9 ±3.6 vessels mm^−2^, *p* > 0.05, n.s.). However, there was a substantial difference in the mean blood vessel size. This was represented as the percent vasculature observed in each imaged section of regenerated tissue (Figure 1D). The TCM group demonstrated 2.40% ± 0.51 vascularization while the control group showed 1.14% ± 0.37 vascularization (*p* < 0.05). This indicates that while the number of vessels in the regenerated regions are similar in bladders instilled with either TCM or media, the size of the vessels are much larger and prominent in tissues instilled with TCM. As an adjunct, immunofluorescence co-staining of von Willebrand factor (vWF) and CD31 (Figure 1E), which are endothelial cell markers seen in vascular beds in human tissue, demonstrated higher global expression in the TCM group compared to the media control. Taken together, these findings demonstrate overall greater revascularization in animals instilled with TCM.

### 2.2. Muscle and Urothelial Quantification

Masson’s trichrome staining was used to assess muscle-to-collagen ratios in all regenerated tissue (Figure 1A). Previous data have demonstrated that unseeded grafts of athymic nude rats contained 20% muscle 4 weeks post-augmentation.^[6]^ In the present study, TCM-instilled animals displayed 52.2% ± 7.7 muscle/collagen content while control animals had 34.3% ± 6.9 (*p* < 0.05) (Figure 1C). Normal muscle/collagen ratios in the rat are ≈1:1. Immunofluorescence co-staining of Caldesmon + Smooth muscle myosin heavy chain (SMMHC) and Calponin + SMMHC demonstrated greater overall expression of smooth muscle-related proteins in TCM regenerated tissue when compared to graft tissue from the control group (Figure 1E). Urothelium width (μm) was measured using Masson’s trichrome-stained images. Urothelium width measurements were made from the basal layer of the urothelium to its apical layer from multiple aspects of the regenerated bladder tissue. Rats have a normal urothelium width that ranges from 50 to 70 μm. The data demonstrated that TCM animals maintained a mean width of 76.6 ± 7.4 μm while control animals had a mean width of 50.1 ± 15.1 μm width (*p* < 0.05) (Figure 1B). This confirms appropriate urothelial cell recruitment to regions of tissue regeneration in the presence of cellular-derived TCM.

### 2.3. Peripheral Nerve Regeneration

**Figure 2.**
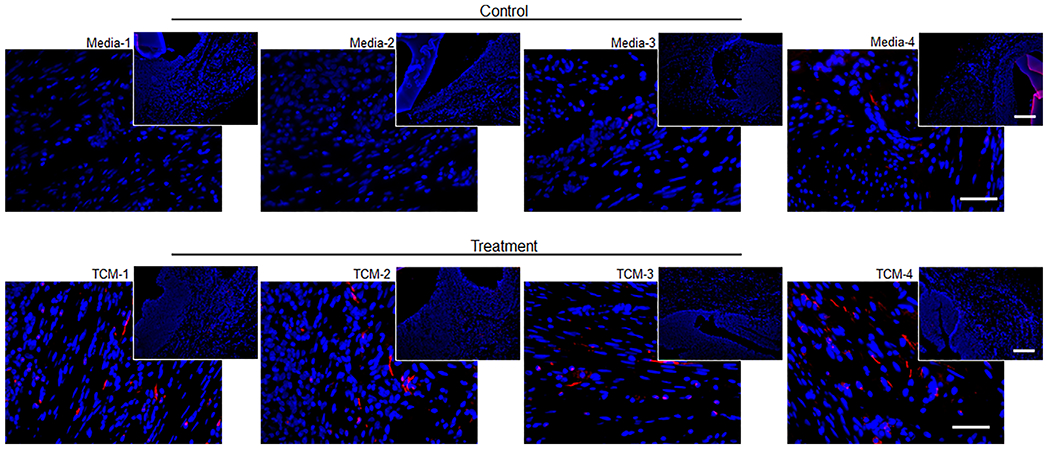
Bladder *β*III tubulin expression. Immunofluorescence staining of bladder tissue for nerve-regeneration related marker, *β*III tubulin. Images depict minimal to no nerve growth in the control animals. Total condition media (TCM) grafts demonstrated significant nerve growth and longer nerve length in all regions of the graft. Inset images 10X scale bar set to 500 μm, 40X Scale bars represent 50 μm.

To determine the effect of TCM instillation on bladder peripheral nerve regeneration, explanted native and regenerated bladder tissue areas were stained with neuronal-specific antibody *β*III tubulin (Figure 2). All five TCM-instilled animals revealed nerve infiltration on all sides of the graft at 4 weeks post-augmentation, while only two of four control animals demonstrated minimal neuronal growth on one side of each graft (Table 1). The maximum average nerve regeneration distance in the TCM-treated group (*n* = 5 demonstrating nerve growth) was 1000.5 ± 238.7 μm versus 460.8 ± 71.8 μm in the control group (*n* = 2 demonstrating nerve growth) (*p* < 0.001). The mean nerve length in the TCM group was 41.2 ± 7.0 μm versus 25.2 ± 8.7 μm in the control group (*p* < 0.05). These findings clearly demonstrate that TCM reproducibly promotes neural growth and infiltration in regenerating bladder tissue, which has the potential to allow for complete innervation of the graft.

### 2.4. Bladder and Kidney Evaluation Following Bladder Augmentation

**Figure 3.**
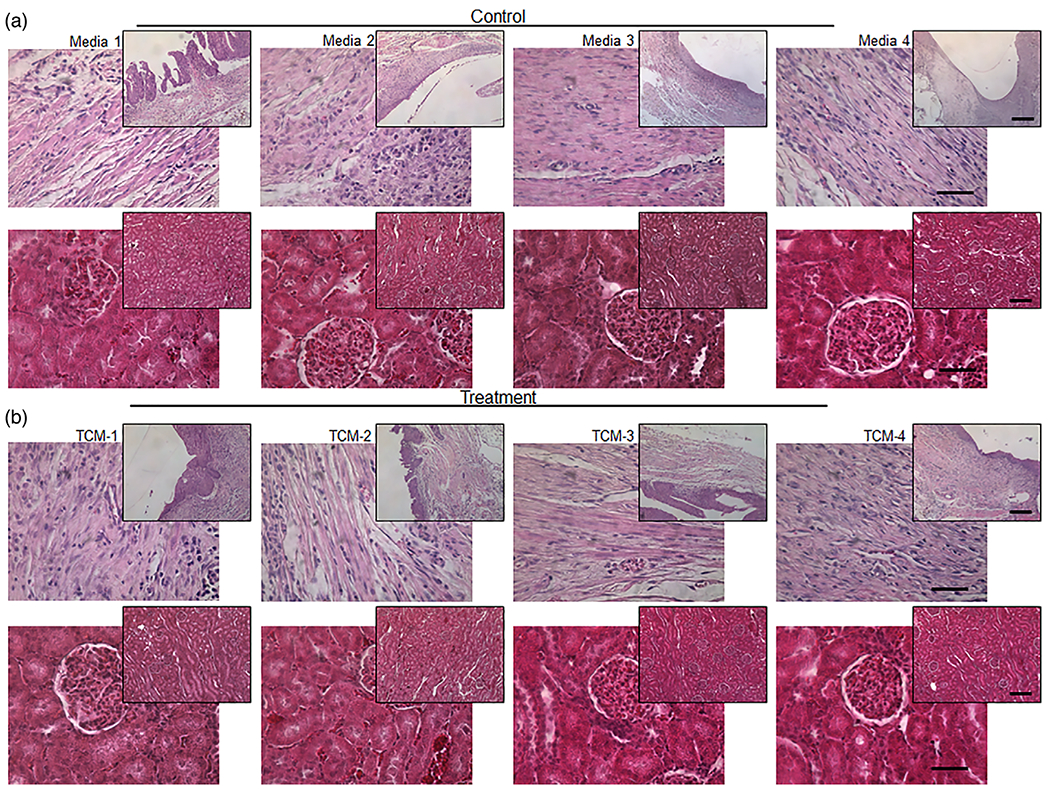
H&E staining of bladder and kidney tissue 4 weeks post-augmentation. At 4 weeks post-augmentation, animals were euthanized and bladders and kidneys were harvested, processed, and stained with Hematoxylin and Eosin (H&E). A) Images of bladder tissue (first row) demonstrate normal urothelial and smooth muscle architecture in media-instilled control animals. Images of kidney tissue (second row) demonstrate normal architecture with no evidence of hydronephrosis microscopically in media-instilled control animals. B) Images of bladder tissue (third row) demonstrate normal urothelial and smooth muscle architecture in Total condition media (TCM) animals. Images of kidney tissue (fourth row) demonstrate normal architecture with no evidence of hydronephrosis microscopically in TCM animals. Inset images 10X scale bar set to 500 μm, 40X Scale bars represent 50 μm.

No bladder or kidney stones were observed on gross inspection of all nine animals (Figure S1, Supporting Information). Kidney morphology appeared normal at the gross and microscopic levels following Hematoxylin and Eosin (H&E) staining (Figure 3A,B). No hydronephrosis or ureteral dilation was noted in all animals 4 weeks post-augmentation (Figure S1, Supporting Information).

### 2.5. Urodynamic Studies

**Figure 4.**
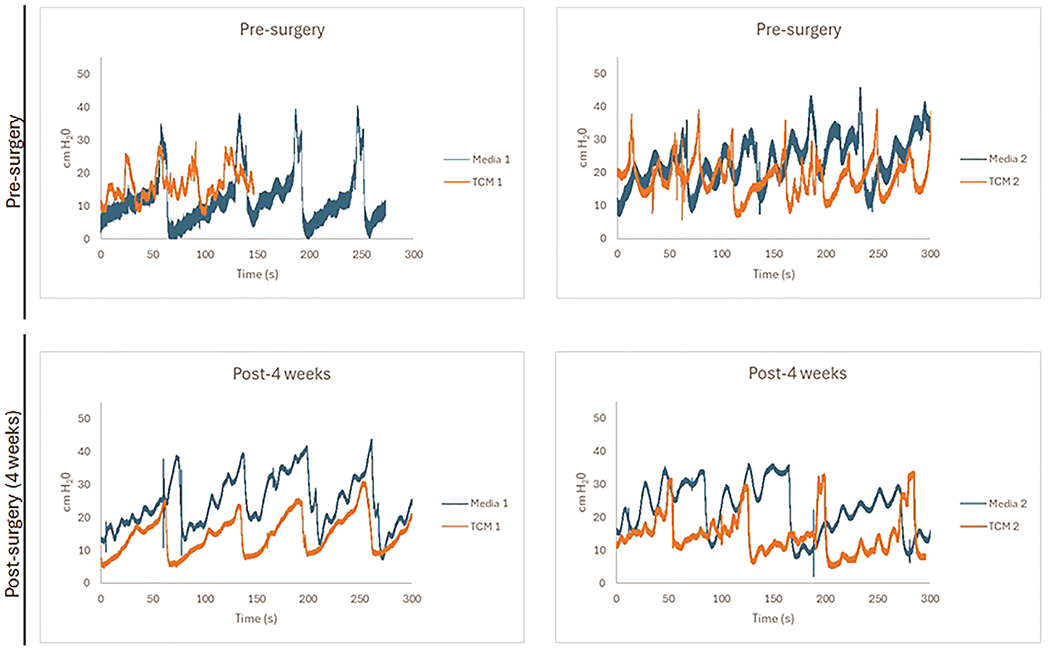
Urodynamic Studies. Urodynamic studies were performed pre- and 4 weeks post-augmentation. Intravesical pressures were directly measured and plotted as a function of time (from 0 to 300 s) during bladder filling in both the control (Media 1 and 2) and Total condition media (TCM) treatment groups (TCM 1 and 2).

Urodynamic studies (UDS) were performed to assess bladder function pre- and post-augmentation for all groups (Figure 4). Prior work from our group has demonstrated a rat bladder compliance (the percentage of bladder volume filled at less than 20 cm H_2_O) greater than 0.50 to be physiologically normal. In the present study at 4 weeks post-augmentation, the TCM group had a mean compliance of 0.71 ± 0.03 compared to 0.51± 0.10 in the control group (*p* < 0.005). Post-augmentation mean bladder capacity was 972.0 ± 51.6 μL in the TCM group and 867.5 ± 62.4 μL in the control (*p* < 0.05). When compared to pre-augmentation capacities, the calculated mean percent bladder recovery (pre-augment capacity: post-augment capacity ratio * 100) was 107.9% ± 4.6% in the TCM group compared to 91.2% ± 3.8% in the control group (*p* < 0.05). Pre-augmentation detrusor overactivity was noted in two animals prior to any surgical intervention or instillation, and therefore the significance of this finding is unknown.

## 3. Discussion

Patients undergoing bladder augmentation enterocystoplasty for the management of lower urinary tract dysfunction often suffer from post-operative complications from the use of intestinal tissue.^[3,5]^ The use of a synthetic scaffold graft (PRS) as a substitute for bowel can prevent many of these long-term complications. In order to mimic the properties of native bladder tissue, all components of the bladder wall must be present including muscle, nerves, urothelial cells, and vasculature to provide adequate tissue perfusion. Significant advancements have been made in the field, whereby engineered biocompatible scaffolds seeded with autologous human bone marrow-derived stem cells can promote tri-layer bladder tissue regeneration, effectively mimicking the biomechanical properties of the native bladder wall, such as expansion and contraction.

Prior work from our group has shown that MSCs combined with CD34+ HSPCs participate in the formation of blood vessels, as well as increased muscle content, peripheral nerve regrowth, and urothelial cell recruitment, when compared to experimental control animals in a nude rat bladder augmentation model as well as in a non-human primate model.^[6–8]^ However, the acquisition of these cells by means of bone marrow aspiration remains a significant challenge in the clinical setting. Bone marrow aspiration is not only costly, but confers procedural risks including infection and postoperative pain. Additionally, the exact number of cells that can be acquired varies with each bone marrow aspiration, and the expansion of these cell populations ex vivo is limited.^[9,10]^ The present study is the first to demonstrate a completely acellular approach, utilizing TCM derived from co-culturing MSCs and CD34+ HSPCs to regenerate bladder tissue in vivo.

Accumulating evidence in the wound-healing literature suggests that stem cells have the ability to aid in tissue regeneration by releasing molecules that exert paracrine effects and mediate cell-to-cell communication. The conditioned media derived from stem cells is believed to contain cell-secreted proteins, cytokines, growth factors, and extracellular matrix as well as microvesicles (MVs), exosomes, and metabolites.^[11]^ A study by Ratajczak et al in 2005 established microvesicles as crucial components to cellular environments, with pleiotropic properties.^[11]^ Xie et al. later demonstrated that MVs (which are packaged with mRNAs, microRNAs, proteins, and lipids) excreted by embryonic stem cells can improve the ex vivo expansion of hematopoietic progenitor cells (HPCs). Various animal models of disease processes leading to tissue injury have shown the therapeutic efficacy of MVs to be comparable to that of the parent stem cells in their ability to reprogram target cells.^[12]^ This study further reported that MVs provide hematopoiesis-supporting effects of MSCs. Results showed that MSC-MVs could promote the expansion CD34^+^-selected cord blood (CB) cells in vitro and generate primitive cells. They concluded that MVs alone may be able to maintain the functional properties of HSPCs and that while MVs were not as potent as the parent cells in their effects, the rate of expansion of CB cells and the number of generated primitive cells were higher when MSC-MVs were added to the co-culture system.^[12]^

In the present study, we evaluate the effects of instilling co-cultured TCM in a rat bladder augmented with an unseeded graft. We note that, compared to instillation of uncultured media alone, there was a statistically significant increase in regeneration of all components of bladder tissue including muscle, urothelial cells, nerves, and blood vessels. This suggests that factors present as part of this “secretome” housed in the TCM may contain properties that feed the cells of the native bladder tissue and promote de novo tissue regrowth throughout the graft. Prior studies focusing on the use of the MSC secretome in wound healing have identified many soluble proteins that contribute to inflammation (ex. IL-6, IL-8), proliferation (ex. EGF, BMP), angiogenesis (ex. VEGF, TGF-b), and remodeling (ex. FGF, MMP-1, ICAM).^[13]^ Notably, MSC-extracellular vesicles (EVs) enhance neovascularization through the stimulation of fibroblast and endothelial cell migration. In a study by Yang et al, the angiogenic property of MSC-EVs was evaluated in an in vivo murine skin wound model.^[14]^ They found that microRNAs such as miR-135b-5p and miR-499a-3p were upregulated and were directly correlated to endothelial cell proliferation in the formation of blood vessels.

The clinical translational literature on cell-free therapies in tissue regeneration is limited but of growing interest in the field of regenerative medicine. This has been of particular interest in the field of cardiac disease.^[15,16]^ A study by Nguyen et al looked at the role of MSCs in promoting myocardial regrowth through paracrine secretory pathways as opposed to stem cell differentiation.^[15]^ This data further supported the hypothesis that a combination of cytokines, chemokines, anti-inflammatory factors, and growth factors direct cell viability, apoptosis, fibrosis, angiogenesis, and proliferation. Another study by Angoulvant et al. demonstrated the use of MSC-conditioned media to reduce myocardial injury in an ex vivo model of isolated rat hearts subjected to ischemia and reperfusion.^[17]^ This data corroborates findings from the hematopoiesis literature suggesting a promising role for stem cell-derived secretory factors in tissue healing and regeneration.

The study presented here is the first to describe an approach to bladder tissue regeneration following unseeded graft augmenttation, by direct instillation of MSC/ CD34+ HSPC co-cultured media. Within the field of bladder tissue engineering, recent work by Zhao et al has described the construction of an artificial acellular nanocomposite scaffold loaded with stromal vascular fraction (SVF), which is released by gradient degradation of the scaffold.^[10]^ In this study, the authors showed that SVF isolated from the secretome of epididymal adipose tissue could promote angiopoiesis and smooth muscle cell proliferation, attenuate inflammation and fibrosis, and improve urodynamic parameters in a rat bladder augmentation model.^[10]^ While their findings were promising, the results from the study concluded superiority in urodynamic parameters of rat bladders that underwent cystotomy alone and were not augmented. This could be due to lower baseline compliance of the ANS material in addition to suboptimal regenerative properties offered by SVF. In contrast, the results of our study demonstrate superiority in all urodynamic parameters following partial cystectomy, augmentation with PRS, and instillation with TCM including increased compliance, increased capacity, and greater percent recovery.

The present study is not without limitations. Given the small sample size, there is a need for additional studies evaluating the regenerative capacity of TCM instillation in larger cohorts of rats undergoing bladder augmentation with unseeded scaffolds. In addition, while regeneration is observed 4 weeks post augmentation, urodynamic studies following instillation at 8 and 12-week time-points would provide further insight into long-term recovery. Future studies in baboon models and clinical trials will require optimization of a treatment course of this novel intravesical therapy to ensure robust functional bladder tissue regeneration.

## 4. Conclusion

Here, we describe the development and acquisition of total condition media derived from co-culture of MSC/CD34+ HSPCs and the use of TCM to regenerate all components of the bladder wall in rats augmented with a pliable synthetic scaffold. Our data indicate that intravesical instillation of TCM promotes tissue regeneration that is reproducible and robust throughout the graft in the absence of cell seeding and results in functional outcomes confirmed by improved urodynamic parameters following augmentation and treatment. Our data suggest that TCM may be used as an alternative to cell-based scaffolds in bladder regeneration and therefore may offer a paradigm shift in the future of bladder augmentation for patients with bladder dysfunction.

## 5. Experimental Section

### PRS [Poly(1,8-Octamethylene-Citrate-Co-Octanol] Scaffold Synthesis:

In order to synthesize poly (1,8-octamethylene-citrate-co- octanol) (PRS), octanol, 1,8- octanediol, and citric acid (Sigma Aldrich, St. Louis, MO) were added to a flask in a 0.2: 0.8: 1 molar ratio and then melted in a silicon oil bath at 165 °C with nitrogen gas flow. The mixture was stirred for ≈15 min. Once the solution was melted, the flask was transferred to another oil bath at 140 °C and subjected to 3 h under nitrogen gas flow forming a pre-polymer. This was dissolved in ethanol and purified by precipitation in Milli-Q water with 20% ethanol, with two additional purification steps in Milli-Q water and final precipitation collection, which was frozen at −80 °C for 12–16 h. The pre-polymer was then lyophilized for 2–3 days until clear and dissolved in 40% ethanol w/v. PRS was characterized using proton nuclear magnetic resonance (1H-NMR, X500, Bruker) and mass spectrometry (Amazon-SL, Bruker).

### Cell Culture:

Human bone marrow MSCs (StemCell Technologies, Vancouver, BC) were thawed and seeded at a density of 4000 viable cells cm^−2^ (100 000 cells / 25 cm^2^ flask) in MesenCult-ACF Plus Medium (StemCell Technologies) for 72 h. Human bone marrow CD34+ HSPCs (StemCell Technologies) were then thawed and co-seeded with the MSCs at a concentration of 4000 viable cells cm^−2^. The cells were cultured in a 1:1 mixture of StemSpan-XF: MesenCult-ACF Plus Medium (Stem Cell Technologies). Cell viability was determined by NucBlue live cell stain (Thermo Fisher Scientific, Waltham, MA) prior to seeding. The co-cultured cell media was changed 24 h prior to each animal bladder instillation. Immediately preceding each bladder instillation, the co-seeded cell populations in each flask were pelleted by centrifugation at a speed of 1000 relative centrifugal force (RCF) for 5 min and fresh supernatant of total condition media (TCM) was collected in preparation for bladder instillations. The pelleted cells were resuspended and plated into the same culture flask in 2 mL of fresh 1:1 StemSpan-XF: MesenCult-ACF Plus Medium. A 1:1 mixture of StemSpan-XF: MesenCult-ACF Plus Medium was prepared for control group bladder instillations.

### In Vivo Bladder Augmentation Studies:

Sprague Dawley rats (females weighing ≈ 300 g; 9–10 weeks of age; Charles River Laboratories, Wilmington, MA) underwent bladder augmentation as previously described by the laboratory.^[6]^ Rats were anesthetized with inhalation of 2% isoflurane. A 1.0 cm midline incision was created with abdominal fascia and abdominal wall musculature exposed with subsequent identification of the urinary bladder. A 70% supratrigonal anterior-to-posterior cystectomy was performed and the defect was augmented with an unseeded PRS scaffold in all rats. In this study, female rats were used in order to decrease risk of urethral irritation and edema during catheterization for each bladder instillation (ten animals were augmented at the start of the study but one animal died following augmentation due to intraperitoneal urine leak; *n* = nine total animals were utilized). Male rats were not included in this study due to their increased urethral lengths and tortuosity, which would limit the ability to effectively perform bi-weekly catheterization and bladder instillation. The bladder was closed with 7–0 polyglactin suture in a watertight manner and subsequently sutured with surrounding omentum. Rats were catheterized with a 20 gauge angiocatheter and 100 uL of either coseeded cell TCM (*n* = 5) or 1:1 media (*n* = 4) was instilled into the bladder. No significant leakage was noted prior to closure of the abdominal wall with 4–0 chromic running suture. The skin was then re-approximated with 9 mm autoclips. Bladder instillations were performed under anesthesia twice a week for the duration of the 4-week study period. Each instillation consisted of increasing volumes (50–100 uL increase per week) of TCM or media determined by volume at which urethral leakage was noted, reaching a final instillation capacity of 400–500 uL. All animal studies guidelines were set forth and approved by the Northwestern University Institutional Animal Care and Use Committee (IACUC) (Protocol number S00003766).

### Tissue Specimen Processing and Staining:

Whole bladders and kidneys were removed from augmented animals following euthanasia 4-weeks post-augmentation. Specimens were fixed in a 10% buffered formalin phosphate and dehydrated with exchanges of graduated ethanol. The samples were then embedded in paraffin and sectioned onto glass slides at a 5 μm thickness which were subsequently deparaffinized with xylenes, graduated ethanol washes, and deionized water. This was followed by an established staining protocol for Masson’s trichrome and independent H&E staining. The specimens were consecutively deparaffinized with xylenes, dehydrated with ethanol changes, and re-hydrated with deionized water. After these steps, slides were stained with H&E, followed by washes with gradient ethanol solutions.^[6,7]^ Following air-drying, a coverslip was placed over the specimen sample and secured with Permaslip (Alban Scientific Inc.). A total of nine bladders underwent tissue analysis from all experimental groups: control (*n* = 4) and TCM (*n* = 5).

### Blood Vessel Quantification in Areas of Bladder Tissue Regeneration:

Sample images stained with Masson’s trichrome were digitized and further characterized using a Nikon Eclipse 50i Microscope (Nikon Inc., Melville, NY). The images were opened with Adobe Photoshop CS3 (Adobe Systems Inc.). The pen tool in Adobe was utilized to quantify vessel numbers based upon *n* = 10 images per graft in areas of regeneration. Individual vessels were manually selected and the image histogram tool was used to acquire pixel density for each vessel. Data is represented as mean number of vessels per mm^2^ (means ± SE).

### Bladder Tissue Muscle Quantification:

In order to quantify muscle and collagen expression, the digitized images of all Masson’s trichrome stained samples (1600 pixels-1200 pixels, bit depth 24) were opened with Adobe Photoshop CS3. As previously described by our group, a two-fold elevation of magenta levels enhanced the contrast of red pixels from blue pixels.^[6,7]^ This was followed by a two-fold depression of cyan levels in the red and magenta spectra. This was enhanced by a two-fold elevation of cyan levels and then a two-fold depression of magenta levels in the cyan and blue spectra. The selection color range tool was used to digitally select the red or blue pixels. The image histogram tool was used to quantify the selected pixels. The muscle-to-collagen ratio was calculated from these values, as previously described.^[6]^ Any images containing red blood cells, debris, urothelial cells, and PRS scaffold were edited to remove these structures and thereby enhance visualization of muscle content. Data was based upon ten images per animal for each group at the 4-week time-point.

### Immunofluorescent Characterization of Augmented Tissues:

Following the aforementioned deparaffinization process, tissue samples were also subjected to immunofluorescent staining. Slides underwent antigen retrieval, which consisted of 15 min of boiling in citrate buffer (0.01 m citrate solution, pH 6.0 with 0.05% Tween-20) and subsequent cooling at room temperature. The slides were blocked for 15 min in bovine serum albumin (BSA, 5 mg mL^−1^) and then incubated at room temperature with the primary antibody (against *β*III tubulin, smooth muscle myosin heavy chain (SMMHC), calponin, von Willebrand factor (vWF) and CD31. After washing with DPBS, slides were incubated for 30 min with either an Alexa Red 555 or FITC conjugated secondary antibody (Molecular Probes, Carlsbad, CA), rinsed with DPBS. Slides were mounted with Vectashield (Vector Laboratories, Burlingame, CA). Primary antibodies were diluted to working concentrations of 1 μg mL^−1^ while secondary antibodies were diluted to concentrations of 1–10 μg mL^−1^. All samples were also stained with 4′,6-diamidino-2-phenylindole (DAPI) to visualize cellular nuclei.

### Kidney and Bladder Evaluation:

The presence or absence of kidney hydronephrosis was noted visually immediately following euthanasia. Kidney and bladder cross sections were stained with H&E and digitally imaged as previously described.^[6]^ Gross and microscopic evaluation of the kidney and bladder cross-sections were performed.

### Urodynamic Studies and Bladder Capacity Evaluation:

Urodynamic studies (UDS) and bladder capacity measurements were performed prior to bladder augmentation and immediately prior to euthanization. Bladder capacity was measured prior to UDS. Bladders were emptied by manual decompression and then catheterized using 20 gauge angiocatheters (Becton Dickinson, Franklin Lakes, NJ) per urethra. Saline was added at a rate of 150 μL min^−1^ until urethral leakage was observed. Sprague Dawley rats were anesthetized as described above, and a lower abdominal incision was made to expose the bladders. A 20-gauge cannula was inserted into the bladder dome. This was connected to the Pump 11 Elite Syringe Pump (Harvard Apparatus, Holliston, MA) and to a physiological pressure transducer (SP844, MEMSCAP), which was then connected to a bridge amplifier (Model FE221; AD Instruments, Colorado Springs, CO), which plotted continuous readings of the transvesical pressures using LabChart 7.3 Software (AD Instruments). Compliance was calculated as the percentage of bladder filling at pressures less than 20 cm H_2_O. Leak point pressure was noted to be the maximum pressure attained at terminal contraction.

### Statistical Analysis:

The statistical differences between control and treatment groups for urothelial width, muscle quantification, vessel quantification, bladder capacity, and nerve length were calculated by a two-sample *t*-test. The two-sample *t*-test tests the null hypothesis that the means of each continuous variable measured from both the control and experimental groups were equal and provides a measure of statistical significance (*p*-value).^[9]^ A 95% confidence interval (CI) was utilized to estimate the mean of the obtained data points. A *p*-value < 0.05 was considered statistically significant. Standard error was calculated dividing the standard deviation by the sample size’s square root.

### Ethical Standards:

All animal studies were performed in accordance with guidelines set forth and approved by the Institutional Animal Care and Use Committee (IACUC) at Northwestern University.

## Supplementary Material

SI

## Figures and Tables

**Table 1.** Quantification of nerve regeneration was performed including the percent of total animals in each group with positive staining, average nerve length, and maximum nerve regeneration distance. Data represents means ± SE.

	Percent of animals with *β*III tubulin staining	Average nerve length (μm)	Maximum nerve regeneration distance (μm)
Media control	50% (2/4)	25.16 ± 8.72	460.80 ± 71.84
Total condition media (TCM) treatment	100% (5/5)	41.3 ± 7.03	1000.49 ± 238.67

## Data Availability

The data that support the findings of this study are available from the corresponding author upon reasonable request.
