# Supplementary material for: An Acellular Platform to Drive Urinary Bladder Tissue Regeneration
Source: Adv Ther (Weinh). Author manuscript; Available in PMC 2025 Jan 16. (PMC11737526; doi:10.1002/adtp.202400158)
Supplement: SI [file NIHMS2044966-supplement-SI.pdf]

Supporting Information

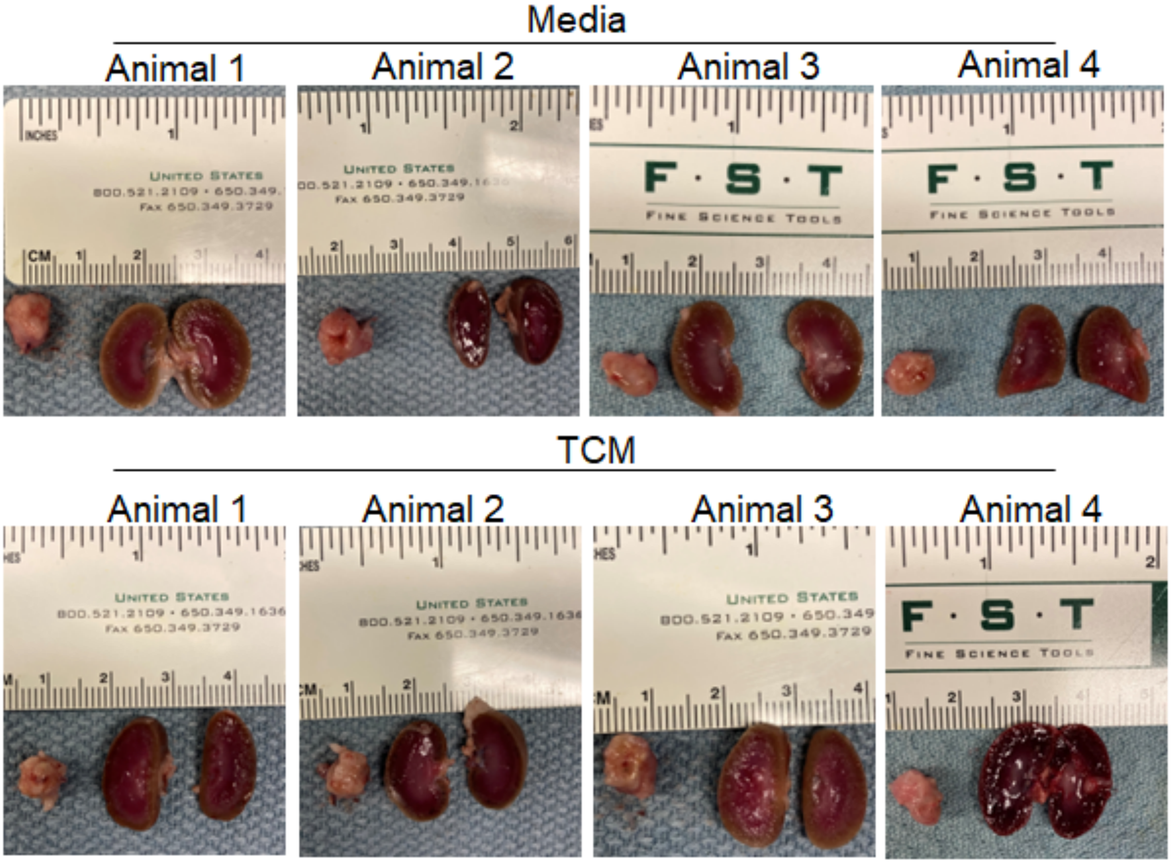

Figure S1. Gross images of bladder and kidneys 4-weeks post augmentation. 4-weeks post-augmentation, animals were euthanized and bladders and kidneys were harvested intact. Images depict no evidence of bladder stone formation or hydronephrosis. All control and treatment group animals displayed similar gross tissue appearance.
